# Urodynamic efficacy of fesoterodine for the treatment of neurogenic detrusor overactivity and/or low compliance bladder

**DOI:** 10.1111/iju.14319

**Published:** 2020-08-07

**Authors:** Kanya Kaga, Tomonori Yamanishi, Mayuko Kaga, Miki Fuse, Tomohiko Kamasako, Mitsuru Ishizuka

**Affiliations:** ^1^ Department of Urology and Surgery Continence Center Dokkyo Medical University Mibu Tochigi Japan

**Keywords:** anticholinergic, detrusor overactivity, fesoterodine, low compliance, neurogenic bladder

## Abstract

**Objective:**

To examine the urodynamic effects of fesoterodine on neurogenic detrusor overactivity and/or low compliance bladder.

**Methods:**

A total of 77 patients (52 men, 25 women; aged 61.6 ± 20.3 years) were given fesoterodine 4–8 mg/day and prospectively followed for 12 weeks. The primary end‐point variable was change in the maximum cystometric capacity on urodynamic study. The secondary end‐point was to assess the number of patients whose neurogenic detrusor overactivity disappeared, and the changes in the urodynamic parameters, lower urinary tract symptoms questionnaires and the 3‐day frequency volume chart parameters after the treatment.

**Results:**

A total of 13 patients (16.9%) withdrew because of adverse events (dry mouth or blurred vision), and four patients dropped out for unknown reasons. Finally, 60 patients completed the study. Bladder capacity at first desire to void, maximum cystometric capacity and bladder compliance increased by 29.2 mL, 79.9 mL and 22.2 mL/cm H_2_O, respectively, showed statistical significance (*P* = 0.026, *P* < 0.001 and *P* < 0.001). Neurogenic detrusor overactivity disappeared in 12 of 51 patients (23.5%), and a significant increase was observed in bladder capacity at first involuntary contraction (*P* < 0.001), and a significant decrease was observed in maximum detrusor contraction (*P* < 0.001). In patients with low compliance bladder (with detrusor underactivity without neurogenic detrusor overactivity; *n* = 9), maximum cystometric capacity and bladder compliance increased significantly (*P* = 0.003 and *P* = 0.006, respectively). Overactive bladder symptom score, International Consultation on Incontinence Questionnaire–Short Form, most items of King’s Health Questionnaire, and the number of urgency episodes and leaks in a day decreased significantly after treatment.

**Conclusions:**

Fesoterodine seems to be a valid treatment option for neurogenic detrusor overactivity and/or low compliance bladder in neurogenic bladder patients.

**Abbreviations & Acronyms**
BVE: bladder voiding efficiency
CIC: clean intermittent self‐catheterization
DO: detrusor overactivity
FDV: first desire to void
FIC: first involuntary contraction
FVC: frequency volume chart
ICIQ‐SF: International Consultation on Incontinence Questionnaire–short form
IPSS: International Prostate Symptom Score
KHQ: King’s Health Questionnaire
LCB: low compliance bladder
MCC: maximum cystometric capacity
NDO: neurogenic detrusor overactivity
OAB: overactive bladder
OABSS: overactive bladder symptom score
Pdet: detrusor pressure
PdetQmax: detrusor pressure at maximum flow rate
PVR: postvoid residual urine volume
Qave: average flow rate
Qmax: maximum flow rate
SD: standard deviation
SF‐36: 36‐Item Short Form Health Survey
UDS: urodynamic study
V‐UDS: video urodynamic study

## Introduction

NDO is recognized when DO is accompanied by a relevant neurological condition.^1^ The main concern regarding patients with neurogenic bladder is renal damage attributable to high detrusor storage and/or voiding pressures. These high detrusor pressures can be caused by DO or LCB. NDO might also cause urinary incontinence and deteriorate quality of life. Thus, the main therapeutic goal for NDO and LCB might be achieving a low‐pressure reservoir and improvement in the patient’s quality of life.^2^, ^3^, ^4^, ^5^

Anticholinergics alone or in combination with CIC is the mainstay therapy for NDO or LCB,^5^, ^6^, ^7^ and assessment with UDS is useful both for the diagnosis and treatment evaluation of NDO.^7^, ^8^ There have been some reports on antimuscarinic drugs for the treatment of NDO or LCB, which reported an increase in bladder capacity and reduction in detrusor pressure, and an improvement in urinary incontinence.^2^, ^3^, ^4^

Previously, we reported the efficacy and safety of tolterodine for the treatment of NDO and/or LCB.^9^ Fesoterodine acts functionally as a prodrug. 5‐HMT is the active metabolite of both tolterodine and fesoterodine, and this active metabolite has been reported to be responsible for the antimuscarinic activity.^10^ The efficacies of fesoterodine for the treatment of idiopathic OAB have been reported.^11^, ^12^, ^13^, ^14^ In the treatment with fesoterodine, flexible dosing strategies involving adjustment of doses of 4 and 8 mg were used to optimize the therapeutic balance between efficacy and tolerability.^11^, ^12^, ^13^, ^14^

The aim of the present study was to investigate the effects of fesoterodine on NDO and/or LCB with neurogenic bladder based on V‐UDS, FVC and lower urinary tract symptom questionnaires.

## Methods

This study was a single‐arm prospective study to evaluate the effects of fesoterodine (4–8 mg/day) on NDO and LCB for 12 weeks. This study was carried out in accordance with the Declaration of Helsinki, registered (UMIN000038269) and was approved by the institutional review board of Dokkyo Medical University, Mibu, Tochigi, Japan (C‐272‐2). All patients signed informed consent before the treatment.

Patients with NDO or LCB in a stable condition for >6 months were included (Table 1). Exclusion criteria were patients having an indwelling catheter, acute urinary tract infection and a history of bladder augmentation. Patients stopped taking medications that might influence voiding function (antimuscarinic drugs, antihistamines, α‐ and β‐adrenoceptor agonists and antagonists) for >2 weeks. After the washout period, the patients received fesoterodine (4 mg/day). The patient‐reported level of satisfaction was evaluated as very satisfied, somewhat satisfied, neither dissatisfied nor satisfied, or dissatisfied, and was assessed at 4 and 12 weeks of treatment.^15^ If the response was favorable, the doses of fesoterodine (4 mg/day) remained the same, and if the patients were not satisfied, the dose of fesoterodine was increased to 8 mg/day after 4 weeks of treatment.^14^

**Table 1:** Patient baseline characteristics

Underlying neurological diseases	*n* (%)
Brain‐related disease	19 (24.7%)
Cerebral infarction	10 (13.0%)
Cerebral hemorrhage	4 (5.2%)
Subarachnoid hemorrhage	1 (1.3%)
Meningioma	1 (1.3%)
Alzheimer’s disease	1 (1.3%)
Normal pressure hydrocephalus	1 (1.3%)
After surgery for chronic subdural blood	1 (1.3%)
Supra‐sacral spine‐related disease	49 (63.6%)
Spinal canal stenosis	20 (26.0%)
Spinal cord injury	12 (15.6%)
Spina bifida	10 (13.0%)
Myelitis	2 (2.6%)
Spinal cord tumor	2 (2.6%)
Spinal cord infarction	1 (1.3%)
Spinal artery malformation	1 (1.3%)
After surgery for epidural abscess	1 (1.3%)
Sacral or peripheral nerve‐related disease	9 (11.7%)
Diabetes mellitus	4 (5.2%)
After surgery for uterine cancer	4 (5.2%)
After surgery for abdominal aortic aneurysm	1 (1.3%)

Urodynamic parameters	Mean ± SD
Bladder capacity at FDV (mL)	154.1 ± 84.9
MCC (mL)	247.0 ± 127.4
Bladder compliance	28.1 ± 30.6
Qmax (mL/s)	10.3 ± 7.5
Pdet at Qmax (cm H_2_O)	43.5 ± 23.2
Bladder outlet obstruction index	25.4 ± 24.4

Total *n* = 77.

Patients recorded a 3‐day FVC, and underwent a V‐UDS at baseline and at month 3. For patients who could void, free uroflowmetry was carried out before and after the therapy. PVR was measured by ultrasonography and BVE (%) = voided volume / (PVR + voided volume) × 100% was calculated. On V‐UDS, a 6‐Fr two‐way catheter was inserted transurethrally, and cystometrogram was recorded at an infusion rate of 50 mL/min. Methods, definitions and units conformed to the standards recommended by the International Continence Society, except where specifically noted.^1^ NDO was defined as “urodynamic observation of involuntary detrusor contractions during the filling phase due to a relevant neurogenic condition.”^5^ Maximum detrusor pressure was calculated as the maximum DO contraction or maximum pressure at the end of the filling phase. Bladder volume at FIC was calculated, but if DO disappeared after the therapy, FIC was evaluated as the MCC.^9^ If patients had no bladder sensation, instillation of fluid was stopped when leakage occurred or detrusor pressure exceeded 60 cm H_2_O.^9^ Under these conditions, this volume was considered as FDV and MCC.^9^ We defined LCB as bladder compliance of ≤20 mL/cm H_2_O.^16^ If patients could void, a pressure/flow study was carried out in a standing or sitting position.^17^

The primary end‐point was change from baseline to the end of treatment in the MCC in V‐UDS. The secondary end‐points were the number of patients whose NDO disappeared, and changes in the following parameters from baseline to the post‐treatment: bladder capacity at FDV, bladder capacity at FIC, maximum detrusor pressure and bladder compliance. If patients could void, Qmax, maximum detrusor pressure and PdetQmax were evaluated. Changes in OABSS, IPSS, ICIQ‐SF and KHQ,^18^, ^19^, ^20^ and the changes in the number and the amount of voids, and the number of daily incontinence episodes in a 3‐day FVC were evaluated from baseline to 4 and 12 weeks of treatment. In the SF‐36, domains of physical functioning, physical role, body pain, general health, vitality, social functioning, role emotional and mental health were evaluated, with a minimum score of 0 (worst health), and the maximum score of 100 (best health).

Adverse events were monitored throughout the study. Changes in PVR and BVE% from baseline to week 12 were calculated.

### Statistical analysis

Data are expressed as the mean ± SD. Statistical significance of changes in parameters between baseline and after the therapy was assessed using the Wilcoxon matched pairs signed‐rank test. The level of *P* < 0.05 was considered to show statistical significance. In the previous study with tolterodine, the sample size was set as 45 patients, based on the ability to detect the deference of 65 mL of MCC at week 12.^9^ In the results of the study, MCC increased by >50 mL in 49% of patients. We considered that 60 patients would yield 80% power to detect such a difference, assuming a SD of 125 mL and α‐error of 0.05. Assuming that approximately 20% of the patients would drop out, 77 patients were considered to be required.

## Results

Data of 77 patients (52 men, 25 women; aged 61.6 ± 20.3 years) were analyzed. NDO was noted in 67 patients, and LCB without detrusor contraction in 10 patients. A total of 31 patients were on CIC. The background characteristics of patients, including urodynamic findings, and total scores of OABSS, ICIQ‐SF and IPSS are shown in Table 1.

A total of 13 patients (16.9%) dropped out because of adverse events, and four patients dropped out for unknown reasons. Finally, 60 patients completed the study. The background of the group that completed UDS before and after the treatment is shown in Table 2. There were no patients with dementia. The dose of fesoterodine was increased in 20 patients (supra‐pontine four patients, supra‐sacral 12 patients and infrasacral four patients), and decreased in no patients.

**Table 2:** Characteristics of the subgroup of patients in terms of the level of injured spinal cord that completed UDS before and after the treatment

	*n* (%)	Mean age	Male/female	CIC	*n* (fesoterodine 8 mg)
Supra‐pontine	15 (25%)	72.9 ± 14.1	10/5	1	4
Supra‐sacral	39 (65%)	50.4 ± 20.6	26/13	24	12
Infra‐sacral	6 (10%)	55.7 ± 14.5	2/4	3	4

Total *n* = 60.

In V‐UDS, bladder capacity at FDV, MCC and bladder compliance increased by 29.2 mL, 79.9 mL and 22.2 mL/cm H_2_O, respectively, showing statistical significance (*P* = 0.026, *P* < 0.001 and *P* < 0.001). In patients with NDO (*n* = 51), DO disappeared in 12 patients (23.5%). Bladder capacity at FDV, MCC and FIC, and bladder compliance increased significantly (*P* = 0.038, *P* < 0.001, *P* < 0.001 and *P* = 0.016) by 26.0 mL, 74.3 mL, 85.5 mL and 25.5 mL/cm H_2_O, respectively. Furthermore, maximum detrusor pressure decreased significantly (*P* < 0.001) by 15.1 cm H_2_O (Table 3). In nine patients with LCB with detrusor underactivity and without NDO, MCC and bladder compliance were increased significantly (*P* = 0.003 and *P* = 0.006, respectively; Table 3).

**Table 3:** Urodynamic parameters at baseline and at week 12

	Before	After	*P*‐value
All patients (*n* = 60)			
Bladder capacity at FDV (mL)	157.8 ± 87.9	187.0 ± 109.3	0.026
MCC (mL)	246.1 ± 123.46	326.0 ± 127.6	<0.001
Bladder compliance (mL/cm H_2_O)	28.0 ± 31.8	50.2 ± 127.6	<0.001
Patients with NDO (*n* = 51)			
Bladder capacity at FDV (mL)	161.6 ± 73.0	187.6 ± 111.3	0.038
MCC (mL)	256.2 ± 116.0	330.5 ± 123.6	<0.001
Bladder compliance (mL/cm H_2_O)	32.3 ± 32.7	57.8 ± 92.5	0.016
Bladder capacity at FIC (mL)	203.9 ± 126.3	289.4 ± 131.8	<0.001
Amplitude of NDO (cm H_2_O)	48.1 ± 22.2	33.0 ± 26.6	<0.001
Free uroflowmetry (*n* = 28)			
Voided volume (mL)	149.2 ± 137.3	145.9 ± 141.7	0.942
Qave (mL/s)	9.3 ± 5.9	8.8 ± 8.3	0.303
Qmax (mL/s)	15.4 ± 10.8	14.7 ± 14.6	0.974
PVR (mL)	14.5 ± 19.2	35.5 ± 38.9	0.008
BVE (%)	85.5 ± 26.8	72.7 ± 29.6	0.190
Pressure/flow study (*n* = 32)			
Qmax (mL/s)	11.5 ± 8.4	13.8 ± 9.7	0.033
Pdet at Qmax (cm H_2_O)	38.7 ± 21.0	36.6 ± 20.7	0.299
Watt factor at Qmax	8.3 ± 3.7	10.0 ± 7.7	0.387
Bladder outlet obstruction index	15.7 ± 29.5	9.0 ± 31.4	0.058
Male (*n* = 18)			
Qmax (mL/s)	10.4 ± 6.3	11.1 ± 7.2	0.396
Pdet at Qmax (cm H_2_O)	48.4 ± 19.4	47.0 ± 25.9	0.713
Watt factor at Qmax	9.0 ± 3.5	11.4 ± 9.2	0.545
Bladder outlet obstruction index	27.5 ± 21.8	24.8 ± 29.4	0.296
Female (*n* = 14)			
Qmax (mL/s)	13.4 ± 10.3	16.9 ± 11.9	0.034
Pdet at Qmax (cm H_2_O)	25.9 ± 18.1	23.9 ± 12.0	0.680
Watt factor at Qmax	7.4 ± 3.9	7.6 ± 4.1	0.652
Patients with LCB without DO (*n* = 9)			
Bladder capacity at FDV (mL)	136.4 ± 151.9	183.6 ± 103.0	0.385
MCC (mL)	188.7 ± 155.6	300.3 ± 154.0	0.003
Bladder compliance (mL/cm H_2_O)	4.0 ± 2.5	7.0 ± 3.8	0.006

Free uroflowmetry and pressure/flow study were carried out in NDO patients who were able to void, and conducted at baseline and at week 12. Qave and Qmax in free uroflowmetry did not change significantly, but PVR increased significantly. No significant changes were observed in pressure/flow study, except for Qmax (Table 3).

On X‐ray images, vesicoureteral reflux was noted in two patients, and bladder deformity was noted in 40 of 50 patients tested; Ogawa’s deformity grade was 3 in four patients, 2 in 15 patients and 1 in 21 patients. Vesicoureteral reflux did not disappear, but de novo vesicoureteral reflux was found in one patient after the 12 weeks of treatment. Bladder deformity did not seem to be changed in the short duration of treatment.

**Fig. 1:**
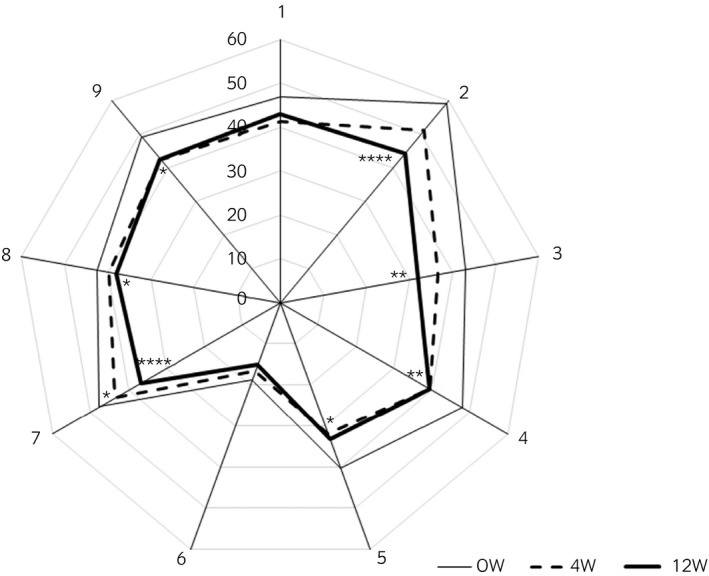
Results of KHQ at baseline (straight line), and at weeks 4 (dotted line) and 12 (bold line; *n* = 49). Numbers on the axis of the radar chart indicate the following domains: 1. General Health Perceptions; 2. Incontinence Impact; 3. Role Limitations; 4. Physical Limitations; 5. Social Limitations; 6. Personal Relationships; 7. Emotion problems; 8. Sleep and Energy; and 9. Severity (Coping) Measures. *P < 0.05, **P < 0.01, and ****P < 0.0001 versus baseline (Wilcoxon matched pairs signed‐rank test).

**Fig. 2:**
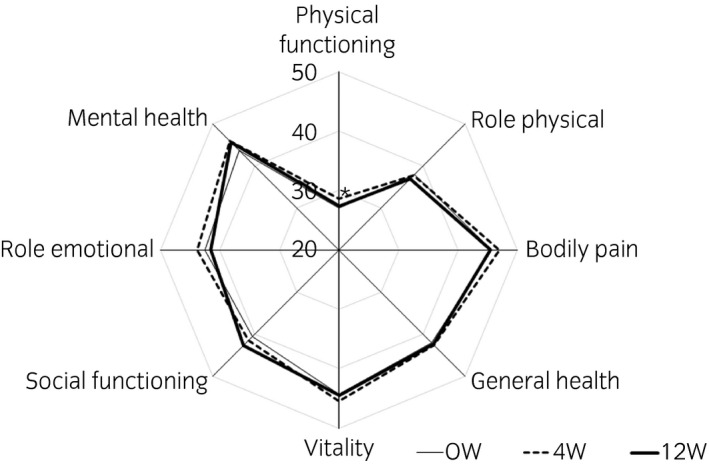
Results of SF‐36 at baseline (straight line), and at weeks 4 (dotted line) and 12 (bold line; *n* = 49). *P < 0.05 versus baseline (Wilcoxon matched pairs signed‐rank test).

The total scores of OABSS and ICIQ‐SF decreased at weeks 4 and 12, respectively (Table 4). In the FVC (*n* = 47), the number of daytime voids, number of urgency episodes, number of leaks and number of pad changes in 24 h decreased significantly (Table 4). Furthermore, the mean voided volume increased significantly. In KHQ (*n* = 49), scores of Incontinence Impact, Role Limitations, Physical Limitations, Social Limitations, Emotions and Severity Measures decreased significantly (Fig. 1). However, all items of the SF‐36 at 12 weeks did not change significantly (*n* = 49; Fig. 2).

**Table 4:** OABSS, ICIQ‐SF, and IPSS at baseline and at weeks 4 and 12

	Before	At 4 weeks	*P*‐value	At 12 weeks	*P*‐value	4 mg at 12 weeks	8 mg at 12 weeks
OABSS (*n* = 55)							(*n* = 19)
Total score	6.7 ± 4.1	5.6 ± 4.0	0.050	5.3 ± 4.0	0.006	4.9 ± 3.8	6.2 ± 4.2
Frequency score	0.7 ± 0.6	0.6 ± 0.6	0.109	0.6 ± 0.6	0.135	0.6 ± 0.5	0.7 ± 0.7
Nocturia score	1.4 ± 1.1	1.3 ± 1.1	0.626	1.2 ± 1.1	0.172	1.2 ± 1.1	1.2 ± 1.2
Urgency score	2.5 ± 1.7	2.1 ± 1.7	0.089	1.9 ± 1.7	0.011	1.8 ± 1.6	2.2 ± 1.7
Urgency incontinence score	2.1 ± 1.8	1.6 ± 1.8	0.144	1.6 ± 1.8	0.066	1.3 ± 1.6	2.1 ± 1.9
ICIQ‐SF (*n* = 55)							*n* = 19
Total score	9.1 ± 5.4	7.6 ± 5.8	0.055	6.9 ± 5.5	<0.001	6.0 ± 5.0	8.9 ± 5.6
Frequency of leaks score	2.5 ± 1.6	1.9 ± 1.7	0.003	1.9 ± 1.7	<0.001	1.7 ± 1.6	2.5 ± 1.6
Amount of leaks score	2.8 ± 1.7	2.3 ± 1.7	0.102	1.9 ± 1.5	<0.001	1.7 ± 1.4	2.3 ± 1.5
Quality of life score	3.8 ± 3.0	3.5 ± 3.2	0.690	3.1 ± 3.0	0.033	2.8 ± 2.7	4.2 ± 3.1
IPSS (*n* = 54)							*n* = 19
Total score	10.0 ± 8.6	8.2 ± 7.9	0.133	8.5 ± 8.2	0.110	5.8 ± 7.0	12.1 ± 8.6
Storage subscore	5.8 ± 4.3	4.7 ± 3.9	0.141	4.5 ± 3.5	0.068	3.7 ± 2.9	5.7 ± 3.9
Voiding subscore	4.0 ± 4.6	3.3 ± 4.2	0.083	3.4 ± 4.4	0.266	2.3 ± 3.9	5.1 ± 4.6
FVC (*n* = 47)							*n* = 20
No. voids/day time	7.5 ± 2.8	6.6 ± 2.6	0.043	6.7 ± 2.2	0.011	6.5 ± 1.9	7.0 ± 2.5
No. voids/night	1.3 ± 1.4	1.1 ± 1.2	0.322	1.1 ± 1.1	0.412	1.1 ± 1.2	1.0 ± 1.0
No. urgency episodes/24 h	2.6 ± 5.3	1.4 ± 2.8	0.061	1.3 ± 2.7	0.032	1.0 ± 1.8	1.8 ± 3.5
No. leaks/24 h	1.5 ± 1.6	1.1 ± 2.1	0.011	1.0 ± 1.9	0.006	0.6 ± 1.4	1.4 ± 2.4
Amount of leaks/24 h (mL)	124.7 ± 191.4	97.3 ± 238.6	0.311	92.0 ± 198.1	0.425	53.0 ± 161.1	143.3 ± 223.7
No. pad changes/24 h	1.4 ± 1.6	1.1 ± 2.1	0.007	1.0 ± 2.0	0.010	0.7 ± 1.5	1.4 ± 2.4
Mean voided volume (mL)	166.6 ± 81.5	198.7 ± 94.0	0.044	197.6 ± 98.2	0.011	208.5 ± 107.8	180.8 ± 73.6
Max voided volume (mL)	286.6 ± 133.5	324.2 ± 149.6	0.101	311.9 ± 132.6	0.365	320.4 ± 138.5	300.0 ± 119.0

Patient‐reported level of satisfaction at 12 weeks of treatment was evaluated as “very satisfied” by 18 patients (30.0%), “satisfied” by two patients (3.3%), “somewhat satisfied” by 19 patients (31.7%) and “neither dissatisfied nor satisfied” by 21 patients (35.0%).

Adverse events were observed in 40 patients (51.9%): dry mouth (*n* = 20), constipation (*n* = 7), blurred vision (*n* = 3) and others in 10 patients (voiding difficulty, urinary retention, headache, sleepiness, urethral discomfort, urgency, nausea, shivering and reflux esophagitis; Table 5). These events occurred after 4 mg/day fesoterodine treatment in 32 patients (80.0%), and 8 mg/day in eight patients (20.0%). Temporary urinary retention was noted in one patient who recovered micturition after stopping the medication.

**Table 5:** Adverse events

	*n* (%)	Total, *n* (fesoterodine 8 mg)
Dry mouth	20 (26.0%)	5
Constipation	7 (9.1%)	2
Blurred vision	3 (3.9%)	1
Voiding difficulty	2 (2.6%)	0
Urinary retention	1 (1.3%)	0
Headache	1 (1.3%)	0
Sleepiness	1 (1.3%)	0
Urethral discomfort	1 (1.3%)	0
Urgency	1 (1.3%)	0
Nausea	1 (1.3%)	0
Shivering	1 (1.3%)	0
Reflux esophagitis	1 (1.3%)	0

## Discussion

In the present study, bladder capacity at FDV, MCC and bladder compliance significantly increased by 29.2 mL, 79.9 mL and 22.2 mL/cm H_2_O, respectively (*P* = 0.026, *P* < 0.001 and *P* < 0.001), after fesoterodine treatment. NDO disappeared in 23.5% of patients with NDO. Bladder capacity at FIC increased significantly (*P* < 0.001), and maximum detrusor pressure decreased significantly (*P* < 0.001). In patients whose DO disappeared after fesoterodine treatment, we could not calculate the changes in FIC. Thus, we calculated FIC as equal to MCC to evaluate changes in these patients.^9^

The OABSS, ICIQ‐SF, most items of the KHQ, and the number of urgency episodes and leaks in a day decreased significantly after treatment. Therefore, fesoterodine seemed to be effective for the treatment of NDO and/or LCB in increasing bladder capacity and bladder compliance. These results seemed to be pronounced in patients with NDO.

In patients with LCB without detrusor contraction, bladder compliance was not normalized after fesoterodine treatment, although it increased with statistical significance. However, MCC increased from the baseline (188.7 mL) to the post‐treatment value (300.3 mL). Therefore, we suppose that the treatment might be meaningful. For the treatment of NDO or LCB, double dosages of antimuscarinic drugs have been reported to be necessary compared with those for patients with idiopathic OAB, which in turn might lead to more severe adverse events and, consequently, to termination of treatment.^4^, ^16^, ^21^ The dose of fesoterodine can be increased to 8 mg flexibly, with relative tolerability. The superiority of fesoterodine 8 mg over fesoterodine 4 mg, and that over tolterodine 4 mg in OAB patients, has been reported.^12^, ^13^ In the present study, fesoterodine 4 or 8 mg was administered as a flexible‐dose plan, with voluntary dose escalation depending on the patient’s satisfaction and tolerability, and 33% of patients took fesoterodine 8 mg/day. Although we did not compare the effect of two doses, the flexible‐dose plan in the present study seemed more efficacious than the fixed dose. The adverse events occurred mostly with the 4 mg/day dosage; thus, increasing dosage to 8 mg/day seemed to be tolerable.

Fesoterodine is not approved for neurogenic bladder. Some patients without urgency or bladder sensation cannot be precisely diagnosed as OAB. However, many kinds of anticholinergic drugs and even β3‐agonists have been prescribed to patients with neurogenic bladder without urgency, or even patients with only urinary frequency or nocturia without urgency. The Japanese OAB guideline categorized OAB as neurogenic OAB and non‐neurogenic OAB, and the former included spinal cord injury. Therefore, anticholinergics, including fesoterodine, can be prescribed for neurogenic bladder patients with NDO. We also explained to the institutional review board of our institution and obtained approval for use in these patients.

Previously, we studied the effects of 4 mg/day tolterodine ER in 46 patients with NDO or LCB. Bladder volume at FDV and MCC increased by 36.8 and 82.3 mL, both showing statistical significance (*P* = 0.0402 and *P* < 0.0001). NDO disappeared in three of 32 patients with NDO; bladder capacity at FIC showed a significant increase (*P* = 0.0009), and maximum detrusor pressure showed a significant decrease (*P* = 0.0025). However, bladder compliance did not increase significantly.^9^ We could not directly compare these results between fesoterodine and tolterodine, but the improvements in urodynamic parameters might be comparable in the two treatment groups. However, the number of patients whose NDO disappeared was higher after the fesoterodine treatment (23.5%) than the tolterodine treatment (9%).

OAB symptoms and incontinence also improved after fesoterodine treatment. OABSS, ICIQ‐SF and KHQ decreased both at 4 weeks and at 12 weeks after fesoterodine therapy. SF‐36 domains, such as physical functioning, physical role, body pain, general health, vitality, social functioning, role emotional and mental health, did not change significantly at 12 weeks. The possible reasons could be that patients with neurogenic disorders might still be suffering in general health domains, and quality of life might not relate to the improvement in lower urinary tract dysfunction.

Mild adverse events were noted in 51.9%, and 16.9% of patients who dropped out due to adverse events, including one patient with urinary retention. These events occurred in 80.0% of the patients after fesoterodine 4 mg/day, and in 20.0% of those after 8 mg. The adverse events were like the reported adverse events in idiopathic OAB patients.^11^ A total of 20 patients (33.3%) were “very satisfied” or “satisfied,” and 19 (31.7%) were “somewhat satisfied” after fesoterodine treatment. Consequently, fesoterodine appeared to be effective and tolerable in patients with NDO, and in those with LCB.

A limitation of the present study was that this was a non‐controlled study, because it was difficult to recruit enough neurogenic patients to provide controls in this study, and we could not obtain approval from the institutional review board to use a placebo in these patients for ethical reasons. The infusion rate of 50 mL/min seemed a little higher in some patients with NDO or LCB. As we used an infusion rate of 50 mL/min in a routine urodynamic study, we did not change it. However, the condition of urodynamic study before and after the treatment was the same.

In the present study, five patients (8.3%) had no bladder sensation. FDV cannot be evaluated in patients without bladder sensation. However, if we excluded the amount, we could not evaluate differences. Therefore, we temporarily calculated FDV as an equal amount with MCC for patients without bladder sensation.

Another limitation was that the participants had mixed types of neurogenic bladder. It would be better to carry out subanalysis in a group of causative neurogenic disorders. However, the number of these patients was limited, and it was difficult to analyze between the subgroups.

In conclusion, fesoterodine seemed to be effective for the treatment of NDO and/or LCB.

## Conflict of interest

None declared.
